# Angiotensin II Use in Refractory Multisystem Shock: A Case Report

**DOI:** 10.7759/cureus.3665

**Published:** 2018-11-30

**Authors:** Mohamed Ahmed, Saba Habis, Ahmed Mahmoud, Cedric Rutland, Rasha Saeed

**Affiliations:** 1 Surgery, Riverside Community Hospital / Envision Healthcare, Riverside, USA; 2 Internal Medicine, Riverside Community Hospital / Hospital Corporation of America, Riverside, USA; 3 Internal Medicine, Riverside Community Hospital, Riverside, USA; 4 Surgery, Riverside Community Hospital / University of California, Riverside, USA

**Keywords:** trauma, vasopressors, vasodilatory shock, angiotensin ii

## Abstract

Distributive (vasodilatory) shock is common in patients admitted to the intensive care unit (ICU). Treating distributive shock presents a challenge, especially if a patient is tachyphylactic to commonly used vasopressors. This case report illustrates the use of a newly approved vasopressor in a patient with vasodilatory shock resulting from a motor vehicle injury. A 56-year-old man was brought to our emergency department (ED) hemodynamically unstable requiring aggressive resuscitation. The results of his evaluation were consistent with multisystem trauma for which he required intubation on arrival, and he received multiple units of blood and blood product via transfusion. The patient’s condition declined despite receiving multiple vasopressors in the ICU. A few days after admission, the patient developed ischemic bowel requiring surgical resection. While his chance of survival was believed to be dismal, the use of angiotensin II (ATII) as a last resort proved to be helpful.

## Introduction

Distributive (vasodilatory) shock is frequently encountered in intensive care unit (ICU) patients, and treating distributive shock can be difficult and puzzling, especially if tachyphylaxis to the commonly used vasopressors becomes an issue. Vasopressors are used in patients who remain hypotensive after adequate fluid resuscitation. There has been a paradigm shift in avoiding the use of dopamine in favor of norepinephrine augmented by vasopressin (an antidiuretic hormone) to minimize the dose of norepinephrine. In cases where a refractory shock is associated with poor cardiac output, dobutamine has been found to be useful. Responsiveness to commonly used drugs can decrease over time as a result of tachyphylaxis. This case illustrates the use of a newly approved vasopressor in vasodilatory shock.

## Case presentation

A 56-year-old male pedestrian was brought to our emergency department (ED) after being struck by a car at high speed resulting in an unstable pelvic fracture, massive retroperitoneal bleeding, pulmonary contusion, and traumatic rupture of the diaphragm which was not evident at the time of admission. He was admitted to our Level II trauma center ICU after initial resuscitation in the ED. Renal failure progressed rapidly due to rhabdomyolysis. His early post-injury course was complicated by refractory shock requiring high doses of vasopressors, hypoxic hypercapnia respiratory failure on ventilation support, ischemic colitis, septic shock, cardiogenic shock that required cardioversion on three different occasions, acute renal failure requiring continuous renal replacement therapy, and shocked liver. The patient required multiple visits to the operating room with initial resection of the terminal ileum and right colon, repair of the diaphragmatic hernia, chest tube insertion followed by washout, ileostomy, feeding gastro-jejunostomy tube, and biologic mesh closure.

Despite receiving high-dose norepinephrine, vasopressin, and epinephrine, the patient’s condition continued to deteriorate with a mean arterial pressure <60 mmHg. Angiotensin II (ATII) was given as an infusion starting with 5 ng/kg/minute increments. The max maintenance dose of 15 ng/kg/minute was achieved in three hours reaching our target blood pressure (BP) for the first 24 hours, and was tapered to 10 ng/kg/minute during the next 12 hours and to 5 ng/kg/minutes during the last 12 hours. ATII was completed with no side effects. The patient’s condition dramatically improved, and he was weaned off of vasopressors within three days of the ATII use. He survived his injuries and was referred to acute rehabilitation.

## Discussion

Shock (hypovolemic, obstructive, distributive, and cardiogenic) is a state of tissue and cellular hypoxia due to decreased delivery, increased consumption or inadequate utilization of oxygen. Distributive shock (also known as vasodilatory shock) is the result of severe peripheral vasodilation. Vasodilatory shock is multifactorial and characterized by peripheral vasodilation and preserved or increased cardiac output, high mortality, and limited therapeutic options [1-2]. Vasoconstriction can be achieved by stimulating adrenergic (alpha, beta 1,2) and dopaminergic receptors, the use of calcium sensitizers, and stimulation of angiotensin (AT) receptors (AT1,2). ATII is an endogenous peptide hormone produced in the pulmonary endothelium. In respiratory failure, pulmonary capillary angiotensin-converting enzyme activity will decrease, impairing ATII production.

ATII has a vasoconstrictive effect on the venous and arterial smooth muscle [3], and exerts its effects by binding to specific AT receptors that vary based on the type of cell membrane (e.g., AT-1, AT-2, AT-4 and Mas receptors). The major physiological effects are mediated by AT-1 receptors located in the kidneys, vascular smooth muscle, lung, heart, brain, adrenals, pituitary gland, and liver and relate to maintenance of hemodynamic stability and fluid and electrolyte regulation [2]. During sepsis, down-regulation of both AT-1 and AT-2 receptors will occur, inhibiting catecholamine release by the adrenal medulla and attenuating the responsiveness of BP and aldosterone formation [4].

Sepsis also leads to an imbalance between vascular mediators. Vasodilating factors (e.g., nitric oxide, tumor necrosis factor-α, histamine, kinins, and prostaglandins) are released, whereas the vasoconstrictor response to ATII and catecholamines might decrease [5-6]. Refractory septic shock is associated with impaired microvascular flow and reduced capillary density at sub pressor levels [7]. Other factors can further influence the development of microcirculatory dysfunction, including low tissue oxygen pressure, production of hypoxia-inducible factors, redox, and ATII potential alterations [6]. The encouraging findings of improved macro-circulatory hemodynamics and decreased catecholamine requirements after ATII administration are offset by evidence of microcirculatory dysfunction that could result from high levels of ATII [8]. Intravenous ATII is associated with increased BP in patients with cardiogenic, distributive, and unclassified shock. Further, a role may exist for ATII in restoring circulation in cases of cardiac arrest [9-10].

When septic shock and respiratory failure coexist, failure of the lungs to convert ATI to ATII could be a factor leading to refractory septic shock, and patients might benefit from exogenous ATII [11]. As part of the renin-angiotensin-aldosterone system (RAAS) in which the kidneys are the driving organ, ATII increases efferent renal arteriolar resistance to a level greater than the afferent renal arteriolar resistance, serving to increase the intra glomerular pressure, decrease overall renal blood flow, and augment the filtration fraction [12]. Exogenous ATII prevents renal ischemia through RAAS and improves renal function without decreasing intrarenal oxygenation during septic shock [13]. ATII plays an important role in liver function as well. The liver fails to produce angiotensinogen during refractory shock leading to a potential need for (and benefit of) exogenous ATII [14]. Patients with a higher severity of illness and relative deficiency of intrinsic ATII who received exogenous ATII had improved mortality and infrequent adverse reactions—evidence that ATII is safe for use in patients with vasodilatory shock. ATII can be an effective novel agent that increases BP in refractory multisystem shock and, potentially, overall survival in patients who do not respond to high doses of conventional vasopressors [2-3,15-16].

## Conclusions

The use of ATII had recently shown to be a possible adjunct to other frequently used vasopressors. Synthetic human ATII does not appear to have a contraindication for its use thus far; however, arterial and venous thromboembolic events have been reported. While further trials are warranted to confirm the benefits of ATII, intensivists should consider the use of ATII in hypotensive patients in distributive shock.
